# Computation Offloading Game for Multi-Channel Wireless Sensor Networks

**DOI:** 10.3390/s22228718

**Published:** 2022-11-11

**Authors:** Heng-Cheng Hu, Pi-Chung Wang

**Affiliations:** Department of Computer Science and Engineering, National Chung Hsing University, Taichung 402, Taiwan

**Keywords:** wireless sensor devices, computation offloading, game theory, channel gain, Nash equilibrium

## Abstract

Computation offloading for wireless sensor devices is critical to improve energy efficiency and maintain service delay requirements. However, simultaneous offloadings may cause high interferences to decrease the upload rate and cause additional transmission delay. It is thus intuitive to distribute wireless sensor devices in different channels, but the problem of multi-channel computation offloading is NP-hard. In order to solve this problem efficiently, we formulate the computation offloading decision problem as a decision-making game. Then, we apply the game theory to address the problem of allowing wireless sensor devices to make offloading decisions based on their own interests. In the game theory, not only are the data size of wireless sensor devices and their computation capability considered but the channel gain of each wireless sensor device is also included to improve the transmission rate. The consideration could evenly distribute wireless sensor devices to different channels. We prove that the proposed offloading game is a potential game, where the Nash equilibrium exists in each game after all device states converge. Finally, we extensively evaluate the performance of the proposed algorithm based on simulations. The simulation results demonstrate that our algorithm can reduce the number of iterations to achieve Nash equilibrium by 16%. Moreover, it improves the utilization of each channel to effectively increase the number of successful offloadings and lower the energy consumption of wireless sensor devices.

## 1. Introduction

Wireless sensor devices (WSDs) have emerged in various applications of smart cities, remote healthcare, unmanned aerial vehicle (UAV) and smart homes [1,2,3,4,5], where WSDs generate and transmit remote sensing data to sink nodes. Various types of modern sensor devices may generate a great volume of data [6,7]. For example, mobile devices capable of sensing and computing may act as sensors to participate in crowdsensing for collecting video, images, or voice [8]. These WSDs share data and extract information for operations of common interest. While both energy and computation capabilities of WSDs are usually limited, some environmental monitoring tasks may require low response latency [9,10]. Although the computation capabilities of WSDs are increasing, the energy consumption of WSDs is still problematic for computation-intensive tasks. Therefore, effectively performing computation-intensive tasks is an important challenge for WSDs.

To overcome this challenge, edge computing is a promising and effective approach [11,12]. With edge computing, WSDs could offload their computation tasks which are locally computed originally to remote edge servers with higher computation capabilities through wireless networks. Therefore, the offloading could reduce the energy consumption of WSDs without increasing processing delay.

Although edge computing could increase the efficiency of processing computation tasks for WSDs, the rapidly increasing WSDs may cause radio communication interference to result in longer communication delays when these WSDs offload their tasks to the edge server simultaneously. Currently, multi-channel communication has been applied to several applications of WSDs to enhance the performance of both transmission throughput and energy efficiency [2]. The communication interference can thus be avoided by allocating one channel to each WSD. Unfortunately, this approach is not feasible because the spectrum resources are scarce and expensive. An efficient computation offload policy based on limited spectrum resources is thus desired.

In this paper, we develop an efficient solution for the computational offloading problem based on a multi-channel computation offloading game. Game theory has been widely used to design a decentralized mechanism for decision-making problems. By considering each WSD as a selfish agent, each agent can decide to either perform the computation locally or by the remote edge server according to their own interests. The previous proposals based on game theory [13,14,15,16] only consider computation capabilities and data size for the decision-making. Because of the selfish behavior in game theory, the dynamic channel assignment may result in poor overall performance. For example, when the channel gain of a WSD is particularly large as compared to the other WSDs, the WSD will occupy a channel. In order to avoid this situation, our scheme considers the channel resource allocation problem based on the channel gain of each WSD for the decision-making problem of computation offloading.

The rest of the paper is organized as follows. Section 2 describes related work. In Section 3, we introduce the system model of multi-WSD offloading. Then, we formulate the proposed centralized mechanism and decentralized mechanism for the multi-WSD computation offload problem in Section 4. In Section 5, we introduce the proposed algorithm based on game theory and prove the existence of Nash equilibrium. In Section 6, the results of the simulation are presented according to the proposed mechanism. Finally, the conclusion of this paper is provided in Section 7.

## 2. Related Works

Nowadays, the research on the computation offloading decision problem of multiple wireless devices can be divided into two types, namely centralized and decentralized computation offloading. The mechanisms of centralized computation offloading request all devices to send their statistics data before offloading to edge servers, where the statistics data includes data size, computing capacity, and received signal strength indicator (RSSI). After receiving the data, the edge server yields the optimal solution of resource allocation. A previous work presented a system model of multiple basestations with built-in edge servers to serve wireless devices [17]. This work also considers communication interference and presents an approach based on the genetic algorithm to solve the decision-making problem of energy-efficient offloading. The computation tasks could be divided into subtasks for parallel execution [18]. It is also possible to employ neighboring devices for cooperative partial offloading [19].

In the case that a single device transmits multiple independent computation requests to an edge server, another work determines the order of offloading tasks according to the delay and energy requirements [20]. Zhang et al. proposed a mechanism of energy-efficient offloading for multiple devices [21]. With the decision-making results, the maximum energy consumption of the overall system can be minimized under the time limit of devices. The tradeoff between delay and energy consumption can also be addressed by an iterative search algorithm [22]. This algorithm yields the optimal solution in multi-device environments. Kan et al. formulated the multi-device resource-allocation offloading decision problem and proposed a heuristic algorithm to solve the cost minimization problem [23]. With the technique of energy harvesting, the computation offloading problem for wireless powered WSD networks is also investigated [24,25,26,27,28,29].

The decentralized offloading mechanisms allow each device to make decisions in a distributed manner. Each device can thus decide the most appropriate decision based on its own interests. The offloading decision of each device will affect the decisions of other devices and vice versa. Game theory is one of the most commonly used methods to solve decentralized decision problems. Chen et al. considered the scenario that a single basestation serves multiple devices and proposed a mechanism based on game theory to solve the problem of efficient computation offloading for edge cloud [13]. They also prove the existence of Nash equilibrium. Guo et al. proposed a scenario that multiple devices offload to multiple edge servers [14]. They studied the collaborative computation offloading problem among edge servers. To support the idea of centralized cloud and multi-access edge computing, Guo and Liu proposed a general architecture that devices can choose not only local or edge server but also cloud data centers for computation offloading [15]. Meskar et al. designed a competition game in order to reduce the energy consumption of a device for computation tasks [16]. In this competition game, devices choose the decision with the minimum energy consumption under time constraints according to the decisions of other devices. Yuan et al. extended edge computing to UAV and proposed a Stackelberg game approach for the heterogenous computation offloading decision problem [5]. Recently, techniques of machine learning have been employed for the offloading decision problem [4,30,31].

In the previously mentioned algorithms of decentralized offloading based on game theory, devices make decisions based on their benefits without considering overall system performance. The selfishness may give some devices poor transmission bandwidth and degrade the overall performance of computation offloading. To avoid such situations, we consider the transmission interferences among devices of simultaneously offloading computation tasks and develop a system to achieve balanced offloading performance for all devices.

## 3. System Model

**Figure 1:**
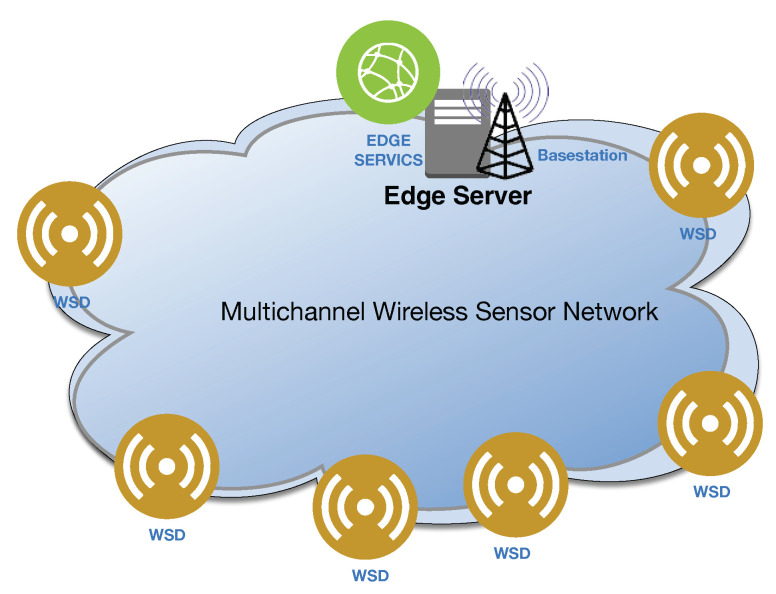
An Illustration of Computation Offloading for WSDs in a Multichannel Wireless Sensor Network.

We present the system model of this work in Figure 1. We consider that a set of *n* WSDs, $N=wsd_{1},wsd_{2},wsd_{3},\ldots ,wsd_{n}$, are distributed in an area, where each WSD needs to complete a computationally intensive task within a limited time period. These WSDs are connected to one basestation through multiple wireless channels. In addition, each basestation is connected to an edge server, which is connected to a power outlet. The edge server has much higher computation capability than WSDs and allows WSDs to offload their tasks. Similar to the previous studies on offloading of cloud computing and edge computing [13,14,15,16,17,20,21,22,23,32,33,34,35,36,37,38], we consider a static scenario, where the states of WSDs do not change during the computation offloading.

### 3.1. Communication Model

Next, the communication model of edge computing is introduced. Each basestation has a set of *m* wireless channels denoted as M={1,2,3,…,m}. Because each channel may suffer from wireless interferences, we use OFDMA to share spectrum resources to multiple WSDs [10]. We make the channel resources of the system orthogonal to each other, but the number of channel resources is not enough to allocate one channel for each WSD. We express $0\leq a_{i}\leq \left|M\right|$ as the computation offloading decision of $wsd_{i}$. Specifically, if $a_{i}>0$, it means that $wsd_{i}$ decides to offload its task through wireless channel $a_{i}$; if $a_{i}=0$, it means that $wsd_{i}$ chooses to compute its task locally. Based on the decision variables $A=(a_{1},a_{2},\ldots ,a_{n})$ of each WSD, we can calculate the uplink data rate $r_{i}$ of $wsd_{i}$ that decides to offload the task to the edge server as follows [39].

(1) $$r_{i}=W\log_{2}\left(1+\frac{p_{i}h_{i,s}}{\bar{\omega_{0}}+\sum_{j\in N:a_{j}=a_{i}}p_{i}h_{i,s}}\right),$$

where *W* is the channel bandwidth, and $p_{i}$ is the transmission power of $wsd_{i}$. $h_{i,s}$ denotes the channel gain between $wsd_{i}$ and the basestation based on the path loss and shadowing. $\bar{w_{0}}$ denotes the background noise power.

### 3.2. Computation Model

Then, we introduce the computation model for both local and edge computing. We assume that each $wsd_{i}$ has a task $J_{i}=\left(B_{i},D_{i}\right)$ to be calculated. Each task can be calculated locally or offloaded to an edge server according to the WSD’s decision. $B_{i}$ indicates the data size of the task $J_{i}$ and $D_{i}$ denotes the number of CPU cycles required to complete task $J_{i}$. Then, we show the processing time and energy consumption required for local computing and edge computing.

**Local Computing**

If $wsd_{i}$ decides to calculate task $J_{i}$ locally, the delay and energy consumption are described below. Let $f_{i}^{l}$ be the local computation capability (i.e., CPU cycles per second) of $wsd_{i}$. The delay time required for local computing is defined as

(2) $$T_{i}^{l}=D_{i}/f_{i}^{l}.$$

The energy consumption of $wsd_{i}$ for the computation is given as

(3) $$E_{i}^{l}=\gamma_{i}D_{i},$$

where $\gamma_{i}$ is the consumed energy per CPU cycle. In order to ensure the completion of computation tasks, we assume that local computing can always meet the delay requirements of computation tasks with higher energy consumption.

**Edge Computing**

We further consider the delay and energy consumption if $wsd_{i}$ chooses to offload its task to the edge server through the basestation. The difference from the local calculation is that the computation offloading requires extra time and energy consumption for transmitting the input data of the task to the edge server. We calculate the transmission time of the offloading input data by using the following equation:

(4) $$T_{i}^{\mathrm{off}}=\frac{B_{i}}{r_{i}}.$$

The transmission energy of $wsd_{i}$ is expressed as

(5) $$E_{i}^{\mathrm{off}}=T_{i}^{\mathrm{off}}p_{i}=\frac{B_{i}}{r_{i}}p_{i}.$$

After the transmission, the edge server performs the computation task $J_{i}$. Let $f_{i}^{m}$ be the computational capability (i.e., CPU cycles per second) of the edge server allocated for $wsd_{i}$. Here, we assume that edge servers always have sufficient resources to fulfill the computing requirements of all WSDs. The delay time required by $wsd_{i}$ to calculate on the edge server is expressed as

(6) $$T_{i}^{c}=\frac{D_{i}}{f_{i}^{m}}.$$

We do not consider the energy consumption of edge servers, because these servers are wire-powered. Thus, the time delay and energy consumption for the edge computation can be expressed as

(7) $$T_{i}^{m}=T_{i}^{\mathrm{off}}+T_{i}^{c}=\frac{B_{i}}{r_{i}}+\frac{D_{i}}{f_{i}^{m}},$$

and

(8) $$E_{i}^{m}=E_{i}^{\mathrm{off}}=\frac{B_{i}}{r_{i}}p_{i}$$

According to Equations (3) and (8), we can obtain the energy consumption for each $wsd_{i}$ as

(9) $$E_{i}=\left\{\begin{array}{cc} E_{i}^{l}, & ifa_{i}=0\\ E_{i}^{m}, & ifa_{i}>0 \end{array}\right.$$

Similar to other works [13,14,15,16,40,41,42,43], we ignore the time of transmitting the results from the edge server to WSDs because the results are usually much smaller than the input data.

Edge computing can reduce the energy consumption of WSDs by offloading computation tasks, but not all WSDs can offload their tasks because of poor channel quality. According to Equation (1), it is necessary to lower channel interference to achieve a higher transmission rate. However, the channel interference is mainly tied to the WSDs sharing the same channel. In order to consider the overall transmission performance, we consider the channel gain of each WSD to reduce channel interferences. Moreover, because a centralized approach may suffer from high system overhead and considerable latency owing to acquiring information from all WSDs, we employ a decentralized approach for the computation offloading decision problem.

## 3.3. Channel Gain

Our main idea is to make WSDs with similar channel gains offload their tasks through the same wireless channel. It is based on the observation that mixing both low-channel-gain and high-channel-gain WSDs in the same channel may result in huge transmission interference to keep these WSDs from successful offloading. Accordingly, we can categorize WSDs into different sets according to their channel gains.

Since we assume that there are *m* wireless channels and *n* WSDs, we can use the k-means to divide WSDs into *m* clusters according to their channel gains. The cluster centers are denoted as $\left\{h_{channel}^{1},h_{channel}^{2},h_{channel}^{3},\ldots ,h_{channel}^{m}\right\}$. They are also equivalent to the channel’s qualities. We use the difference between each WSD’s channel gain and each channel’s quality by calculating the distance from the cluster center based on the following equation:

(10) $$\underset{h_{channel}^{j}}{\arg\min}\;\;\left|h_{i,s}-h_{channel}^{j}\right|.$$

Therefore, we can define the difference between $wsd_{i}$ and channel *j*’s quality as

(11) $${\mathrm{diff}}_{i}\left(j\right)=\left|h_{i,s}-h_{channel}^{j}\right|.$$

According to the above formula, WSDs with similar channel gains will choose the same channel to offload their tasks.

## 4. Problem Formulation

In this section, we separately consider the problem of centralized computing offloading and decentralized computing offloading.

### 4.1. Centralized Edge Computing

In a centralized edge-computing system, we can formulate the energy optimization problem as follows:

(12) $$\begin{array}{cc} \min & \sum_{1\leq i\leq n}E_{i},\\ \; & s.t.\;a_{i}\in \left\{0,1,2,3,\ldots ,M\right\},\\ \; & T\left(a_{i}\right)\leq T_{i}^{max}. \end{array}$$

$T(a_{i})$ is the time spent for $wsd_{i}$ if the WSD chooses the channel $a_{i}$ for task offloading. $T_{i}^{max}$ denotes the maximum allowable delay for $wsd_{i}$.

WSDs transmit their statistics to the edge server to make system-wide decisions in a centralized manner. Although the centralized optimization can minimize overall energy consumption, it is problematic to enforce WSDs to comply with the remote decisions.

### 4.2. Decentralized Edge Computing

In a decentralized edge-computing system, we can formulate the energy optimization problem for each WSD in the following equation:

(13) $$\begin{array}{cc} \min & E_{i}\left(a_{i},a_{-i}\right),\\ \; & s.t.\;a_{i}\in \left\{0,1,2,3,\ldots ,M\right\},\\ \; & T\left(a_{i}\right)\leq T_{i}^{max}. \end{array}$$

where $a_{-i}$ = $\left(a_{1},\ldots ,a_{i-1},a_{i+1},\ldots ,a_{n}\right)$ be the set of decisions of the other WSDs except $wsd_{i}$.

In this system, all WSDs make decisions according to their own interests. Therefore, the decision of each WSD could be selfish for the whole system. Each WSD considers the current decision state of other WSDs before making the best decision for themselves. In this paper, we consider the decision-making problem of distributed computation offloading among the WSDs and present a feasible algorithm.

## 5. Multi-Channel Computation Offloading Game

In this section, we implement the multi-channel computation offloading game for mobile edge computing.

### 5.1. Game Formulation

Let ${-a}_{i}$ be the other decisions that can meet time constraints but are not chosen by $wsd_{i}$. Then, given $a_{-i}$, $wsd_{i}$ would like to set its decision variable $a_{i}$ as the solution of the following equation:

(14) $$\begin{array}{cc} \min & \;E_{i}\left(a_{i},a_{-i}\right),\\ subject\;to & \;a_{i}\in \left\{0,1,2,3,\ldots ,M\right\},\\ \; & T\left(a_{i}\right)\leq T_{i}^{max},\\ \; & \mathrm{diff}\left(a_{i}\right)I_{\left\{a_{i}\neq 0\right\}}\leq \mathrm{diff}\left(-a_{i}\right)I_{\left\{a_{i}\neq 0\right\}}. \end{array}$$

$I_{\left\{A\right\}}$ is an indicator function, where *X* is a conditional expression. If *X* is true, $I_{\left\{X\right\}}$ is equal to 1; otherwise $I_{\left\{X\right\}}$ is equal to 0.

In order to ease the explanation of the problem, we can set the energy consumption function of the problem as the following equation:

(15) $$E_{i}\left(a_{i},a_{-i}\right)=\left\{\begin{array}{cc} E_{i}^{l}, & \mathrm{if}a_{i}=0,\\ E_{i}^{m}, & \mathrm{if}a_{i}>0\;\mathrm{and}\;T_{i}\leq T_{i}^{max},\\ \infty , & \mathrm{if}a_{i}>0\;\mathrm{and}\;T_{i}>T_{i}^{max}. \end{array}\right.$$

Then, we formulate the optimization problem in Equation (14) as a strategic game $\Gamma =(N,{\left\{A_{i}\right\}}_{1\leq i\leq n},$${\left\{E_{i}\right\}}_{1\leq i\leq n})$, where the set of $wsd_{i}$ is the set of the rational players, $A_{i}$ is the strategy set of player *i*, and the energy consumption function $E_{i}\left(a_{i},a_{-i}\right)$ of $wsd_{i}$ is the cost function to be minimized by player *i*. The game Γ is the multi-channel computation offloading game. In the following, we introduce the concept of Nash equilibrium.

**Definition 1.**  A strategy set $a^{*}=\left(a_{1}^{*},\ldots ,a_{n}^{*}\right)$ is a Nash equilibrium for the channel selection computation offloading game when no WSD can reduce its cost by changing its decision, i.e.,

(16) $$E_{i}\left(a_{i}^{*},a_{-i}^{*}\right)<E_{i}\left(a_{i},a_{-i}^{*}\right),\forall a_{i}\in A_{i},1\leq i\leq n.$$

Therefore, according to the definition of Nash equilibrium, we can know that when the game reaches equilibrium, each WSD is in the situation of choosing their own best decision and the game has reached its end.

### 5.2. Game Theory with WSD’s Channel Gain

The pseudo-code of the proposed algorithm is listed in Algorithm 1. Initially, this algorithm assumes that the offloading decisions of all WSDs are local computation, i.e., $a_{i}=0$, for all WSDs. Moreover, each WSD will transmit a pilot signal to the basestation first. After the basestation receives pilot signals from all WSDs, the basestation returns all the received interference information to all WSDs. After each WSD receives the interference information, each device computes the best solution for their DOPT problems in multiple iterations. If an offloading decision of a WSD remains the same, the WSD will not update its decision to the edge server. If the new decision is different from the previous one, the WSD will send an update request message to the edge server to compete for validating its updated decision. This means that for $wsd_{i}$, the cost of updating the decision will be lower than that of maintaining the decision of the previous iteration, where the cost is the energy consumption of $wsd_{i}$.

Then, after the edge server receives the update requests from WSDs, it will randomly select one of the WSDs that transmit update requests. The update request of the selected WSD is validated and a reply of update permission is returned to the WSD. The WSDs whose decisions are not selected by the edge server will not receive the reply message from the edge server and will retain the same decision as the previous iteration. In other words, these WSDs will not update their decisions.

After the selected device updates its decision, the next iteration is performed to generate new decisions by repeating the above actions until no WSD requests for updating their decisions. Namely, the decisions of all WSDs reach the Nash equilibrium. Reaching the Nash Equilibrium means that no WSD can obtain a lower cost by changing its decision. Therefore, all the WSD decisions in this iteration form the solution to our multi-channel computation offloading problem.
**Algorithm 1** Game Theory with WSD’s Channel Gain**Initialization:** The initial computation decisions for all WSDs are $a_{i}\left(0\right)=0$1:**repeat** for each WSD, $wsd_{i}$, and each decision time slot *t* in parallel:2:    Send a pilot signal to the basestation on the selected channel $a_{i}\left(t\right)$3:    Receive the interference information on all channel from the basestation4:    $\Delta_{i}\left(t\right)$← compute the best response solution for (DOPT)5:    **if** $\Delta_{i}\left(t\right)\neq a_{i}\left(t-1\right)$ **then**6:        Send an update request message to the edge server to compete for an update decision7:        **if** there is an update permission message received from the edge server **then**8:           choose the decision $a_{i}\left(t+1\right)=\Delta_{i}\left(t\right)$ for next time slot9:        **else** choose the original decision $a_{i}\left(t+1\right)=a_{i}\left(t\right)$ for next time slot10:        **end if**11:    **else** choose the original decision $a_{i}\left(t+1\right)=a_{i}\left(t\right)$ for next time slot12:    **end if**13:**until** no WSD transmits any update to the edge server

### 5.3. Convergence Analysis

Next, we discuss the convergence of the algorithm, which means that we have to prove the existence of Nash equilibrium for the multi-channel computation offloading game. Before that, we first introduce a convenient property that is helpful for proving the existence of Nash equilibrium.

**Definition 2.**  *A game is said to be a potential game if there is a potential function Φ(a) in the game such that $\forall wsd_{i}\in N,a_{-i}\in A_{-i},a_{i},a_{i}^{\prime }\in A_{i}$,*

(17) $$\begin{array}{cc} E_{i}\left(a_{i}^{\prime },a_{-i}\right) & -E_{i}\left(a_{i},a_{-i}\right)<0\\ \Rightarrow \Phi (a_{i}^{\prime },a_{-i}) & -\Phi (a_{i},a_{-i})<0. \end{array}$$

The potential function is a useful tool for analyzing the equilibrium property of a game, because one of the properties of a potential game is that it must have a Nash equilibrium and the finite improvement property [43]. Next, we show that the multi-channel computation offloading game is a potential game.

**Lemma 1.**  *Given a computation offloading strategy set a, the edge server is beneficial for $wsd_{i}$ if the interference $\mu_{i}\left(a\right)=\sum_{wsd_{j}\in N\backslash \left\{wsd_{i}\right\}:a_{j}=a_{i}}p_{i}h_{i,s}$ received on the selected wireless channel $\mu_{i}>0$ satisfies that $\mu_{i}\left(a\right)\leq Q_{i}$, with the threshold*

$$Q_{i}=\frac{p_{i}h_{i,s}}{2^{\frac{p_{i}B_{i}}{WE_{i}^{l}}}-1}-\bar{\omega_{0}}.$$

**Proof.**  According to Equations (3) and (5), if we want the edge server to be beneficial as compared to local computing for $wsd_{i}$, we have the condition $E_{i}^{m}\left(a\right)\leq E_{i}^{l}$, i.e., $\frac{B_{i}}{r_{i}}p_{i}\leq E_{i}^{l}$. Therefore, we can derive the following equation,

$$r_{i}\geq \frac{B_{i}}{E_{i}^{l}}p_{i}.$$

According to Equation (1), we can obtain that

$$\sum_{wsd_{j}\in N\backslash \left\{wsd_{i}\right\}:a_{j}=a_{i}}p_{i}h_{i,s}\leq \frac{p_{i}h_{i,s}}{2^{\frac{p_{i}B_{i}}{WE_{i}^{l}}}-1}-\bar{\omega_{0}}.$$

□

From Lemma 1, we observe that if the interference received by WSD on the channel is low enough, it is beneficial for the WSD to offload the work to the edge server for computing. Conversely, when the interference on the channel is too high for the WSD, the device should perform local computation.

**Theorem 1.**  *The channel selection computation offloading game is a potential game that must have Nash Equilibrium and the finite improvement property.*

**Proof.**  We first construct the potential equation for the channel selection computation offloading game as

(18) $$\begin{array}{c} \Phi \left(a\right)=\frac{1}{2}\sum_{i\in A}\sum_{j\in A\backslash \{i\}}p_{i}h_{i}p_{j}h_{j}I_{\left\{a_{i}=a_{j}\right\}}I_{\left\{a_{i}>0\right\}}\\ +\sum_{i\in A}p_{i}h_{i}T_{i}I_{\left\{a_{i}=0\right\}}. \end{array}$$

Then, Equation (18) can be equivalently written as the following formula:

(19) $$\begin{array}{cc} \; & \frac{1}{2}\sum_{j\in A\backslash \{k\}}p_{k}h_{k}p_{j}h_{j}I_{\left\{a_{j}=a_{k}\right\}}I_{\left\{a_{k}>0\right\}}\\ \; & +\frac{1}{2}\sum_{i\in A\backslash \{k\}}p_{i}h_{i}p_{k}h_{k}I_{\left\{a_{k}=a_{j}\right\}}I_{\left\{a_{i}>0\right\}}\\ \; & +\frac{1}{2}\sum_{i\in A\backslash \{k\}}\sum_{j\in A\backslash \{i,k\}}p_{i}h_{i}p_{j}h_{j}I_{\left\{a_{i}=a_{j}\right\}}I_{\left\{a_{i}>0\right\}}\\ \; & +p_{k}h_{k}Q_{k}I_{\left\{a_{k}=0\right\}}+\sum_{i\in A\backslash \{k\}}p_{i}h_{i}Q_{i}I_{\left\{a_{j}=0\right\}}. \end{array}$$

Then, because

(20) $$\begin{array}{c} \frac{1}{2}\sum_{i\in A\backslash \{k\}}\sum_{j\in A\backslash \{i,k\}}p_{i}h_{i}p_{j}h_{j}I_{\left\{a_{i}=a_{j}\right\}}I_{\left\{a_{i}>0\right\}}\\ +\sum_{i\in A\backslash \{k\}}p_{i}h_{i}Q_{i}I_{\left\{a_{j}=0\right\}} \end{array}$$

is independent of $wsd_{k}$’s strategies $a_{k}$ and

(21) $$\begin{array}{cc} \; & \sum_{j\in A\backslash \{k\}}p_{k}h_{k}p_{j}h_{j}I_{\left\{a_{j}=a_{k}\right\}}I_{\left\{a_{k}>0\right\}}\\ \; & =\sum_{i\in A\backslash \{k\}}p_{i}h_{i}p_{k}h_{k}I_{\left\{a_{k}=a_{j}\right\}}I_{\left\{a_{i}>0\right\}}, \end{array}$$

we can use Equations (19)–(21) to derive the following result:

(22) $$\begin{array}{cc} \Phi (a) & =\sum_{j\in A\backslash \{k\}}p_{i}h_{i}p_{k}h_{k}I_{\left\{a_{k}=a_{i}\right\}}I_{\left\{a_{i}>0\right\}}+p_{k}h_{k}Q_{k}I_{\left\{a_{k}=0\right\}}\\ \; & +\Xi (a_{A\backslash \{k\}}), \end{array}$$

where $\Xi (a_{A\backslash \{k\}})$ is the Equation (20). Since $wsd_{k}$ update decision $a_{k}$ cannot change $\Xi (a_{A\backslash \{k\}})$, we can omit it in the rest of the proof.

Next, we assume that when $wsd_{k}$ decides to update its decision to reduce its cost function, it will make $E_{k}\left(a_{k}^{\prime },a_{-k}\right)<E_{i}\left(a_{k},a_{-k}\right)$. According to the definition of potential game, the update should also cause $\Phi \left(a_{k}^{\prime },a_{-k}\right)<\Phi \left(a_{i},a_{-i}\right)$ in the potential function. In order to prove the case, we consider three cases, namely case (1): $a_{k}>0$, $a_{k}^{\prime }>0$, case (2): $a_{k}=0$, $a_{k}^{\prime }>0$, and case (3): $a_{k}>0$,$a_{k}^{\prime }=0.$

The first case occurs when $wsd_{k}$’s decision is updated from the wireless channel $a_{k}>0$ to the wireless channel $a_{k}^{\prime }>0$. According to Equation (1), because the function $w\log_{2}x$ is monotonically increasing for *x* and the condition $E_{k}\left(a_{k}^{\prime },a_{-k}\right)<E_{i}\left(a_{k},a_{-k}\right)$ is known, we can obtain the result of this inequality:

(23) $$\sum_{i\in N\backslash \left\{k\right\}:a_{i}=a_{k}^{\prime }}p_{i}h_{i}<\sum_{i\in N\backslash \left\{k\right\}:a_{i}=a_{k}}p_{i}h_{i}.$$

Next, we can know the following result according to Equations (22) and (23), that is,

(24) $$\begin{array}{cc} \; & \Phi \left(a_{k},a_{-k}\right)-\Phi \left(a_{k}^{\prime },a_{-k}\right)\\ \; & =p_{k}h_{k}\sum_{i\in A\backslash \{k\}}p_{i}h_{i}I_{\left\{a_{k}=a_{i}\right\}}I_{\left\{a_{k}>0\right\}}\\ \; & -p_{k}h_{k}\sum_{i\in A\backslash \{k\}}p_{i}h_{i}I_{\left\{a_{k}^{\prime }=a_{i}\right\}}I_{\left\{a_{k}>0\right\}}>0. \end{array}$$

In the second case, it means that the decision of $wsd_{k}$ is updated from a decision of local computation, $a_{k}=0$, to edge computing using a wireless channel $a_{k}^{\prime }>0$. We know that if $wsd_{k}$ selects a wireless channel $a_{k}^{\prime }>0$ for offloading, the interference on the wireless channel $a_{k}^{\prime }$ must be lower than the threshold value of interference, i.e., $\sum_{wsd_{i}\in N\backslash \left\{wsd_{k}\right\}:a_{i}=a_{k}^{\prime }}p_{i}h_{i}<Q_{k}$ and $E_{k}\left(a_{k}^{\prime },a_{-k}\right)<E_{i}\left(a_{k},a_{-k}\right)$. Accordingly, we can derive the following equation:

(25) $$\begin{array}{cc} \; & \Phi \left(a_{k},a_{-k}\right)-\Phi \left(a_{k}^{\prime },a_{-k}\right)\\ \; & =p_{k}h_{k}Q_{k}I_{\left\{a_{k}=0\right\}}\\ \; & -p_{k}h_{k}\sum_{i\in A\backslash \{k\}}p_{i}h_{i}I_{\left\{a_{k}^{\prime }=a_{i}\right\}}I_{\left\{a_{k}>0\right\}}>0. \end{array}$$

In the third case, the decision of $wsd_{k}$ is updated from offloading with the wireless channel $a_{k}>0$ to local computation, $a_{k}^{\prime }=0$. This case could happen under two situations. One is $E_{k}\left(a_{k}^{\prime },a_{-k}\right)<E_{i}\left(a_{k},a_{-k}\right)$, the other is $T_{k}\left(a_{k},a_{-k}\right)>T_{k}^{max}$. For the former situation, because $\sum_{wsd_{i}\in N\backslash \left\{wsd_{k}\right\}:a_{i}=a_{k}^{\prime }}p_{i}h_{i}>Q_{k}$, we can obtain the following equation:

(26) $$\begin{array}{cc} \; & \Phi \left(a_{k},a_{-k}\right)-\Phi \left(a_{k}^{\prime },a_{-k}\right)\\ \; & =p_{k}h_{k}\sum_{i\in A\backslash \{k\}}p_{i}h_{i}I_{\left\{a_{k}=a_{i}\right\}}I_{\left\{a_{k}>0\right\}}\\ \; & -p_{k}h_{k}Q_{k}I_{\left\{a_{k}^{\prime }=0\right\}}>0. \end{array}$$

For the later situation, we can derive that $E_{i}\left(a_{k},a_{-k}\right)≅\infty$ according to Equation (15). Thus, we can also conclude that $E_{k}\left(a_{k}^{\prime },a_{-k}\right)<E_{i}\left(a_{k},a_{-k}\right)$ and $\sum_{wsd_{i}\in N\backslash \left\{wsd_{k}\right\}:a_{i}=a_{k}^{\prime }}p_{i}h_{i}≅\infty >Q_{k}$ This also implies that there will be the same result.

Based on the results of the above three possible updates of decision, we can demonstrate that the multi-channel computation offloading game is a potential game that can reach Nash Equilibrium. □

## 6. Simulation Results

In this section, we show the simulation results of the proposed decentralized multi-channel computing offloading algorithm. Section 6.1 describes the simulation parameters and Section 6.2 presents the simulation results.

### 6.1. Simulation Parameters

In our scenario, we consider a basestation whose coverage is 100 m [14]. There are [30–50] WSDs, namely N = [30, 50], randomly distributed within the coverage [13]. There are five available channels, namely M=5 [13], where the bandwidth of channel, *W*, is 10 MHz [44]. The transmission power *p* is 100 mW [13] and the background noise is $\bar{\omega_{0}}=-100$ dbm [13]. The channel gain is defined as $h_{i,s}=l_{i,s}^{-\alpha }$, where $l_{i,s}$ is the distance from $wsd_{i}$ to the basestation. We set the path loss factor as α=4 [13].

The data generated by WSDs could be of various types and sizes. We randomly generate data size, $B=\left[100,1000\right]\times 10^{3}$ bits [23]. The number of required CPU cycles for computation tasks is also randomly selected, where $D=\left[100,1000\right]\times 10^{6}$ cycles [23]. The computation capacity of a WSD, $f^{l}$, is randomly set within $\left[1.5,2.5\right]\times 10^{9}$ cycles/s [23] and the consumed energy per CPU cycle is γ=0.5 J/gigacycle [17]. The edge computation capacity $f^{m}$ is assigned to each WSD that computes on the edge server as $10\times 10^{9}$ cycles/s [13]. The maximum tolerance time $T_{i}^{max}$ for $wsd_{i}$ is [0.7, 1] sec.

### 6.2. Simulation Results

In order to compare the performance of our algorithm, we also implement several mechanisms as listed below.

**Local**: All WSDs compute their tasks locally.
**Random**: The offloading decisions of all WSDs are random.
**Greedy**: A WSD with a larger difference between edge and local computation has a higher priority for computation offloading.
**Game Theory (GT)**: This approach uses game theory for decision-making without considering channel gains of WSDs.
**Game Theory with Channel Gian (GTCG)**: GTCG minimizes the energy consumption of WSDs by performing the game theory, where the communication quality of each channel is considered. ($-a_{i}$ be the other decisions that can meet time constraints but not chosen by $wsd_{i}$.)
**Channel Pre-Allocation+Game Theory (CPA+GT)**: CPA+GT assigns channels for WSDs based on their channel gains initially. Then, it minimizes energy consumption by applying the game theory. ($-a_{i}$ be the other decisions that are not chosen by $wsd_{i}$.)

**Figure 2:**
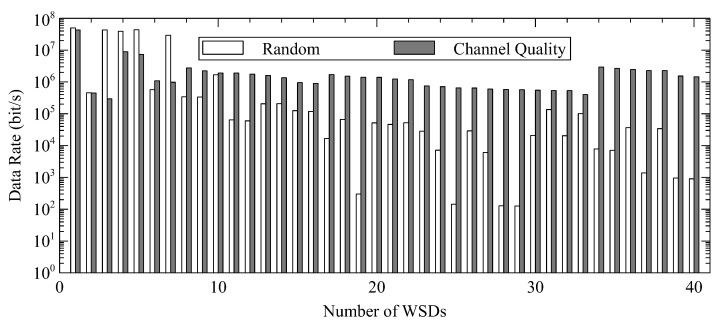
Average Data Rate for WSDs with and without the Proposed Channel Selection Approach.

We first show the effectiveness of the proposed approach of channel selection with 40 WSDs and five channels. Figure 2 shows the data rate of each WSD in descending order of their signal strengths. The data size of each WSD is $500\times 10^{3}$ bits. We can observe that as compared to random channel selection, the selection based on channel quality can improve the data rate of most WSDs. Moreover, the approach can avoid a low data rate because there are only WSDs of similar channel gains in the same channel. By considering channel quality, the transmission rate of most WSDs can be improved to increase the number of successful offloadings.

**Figure 3:**
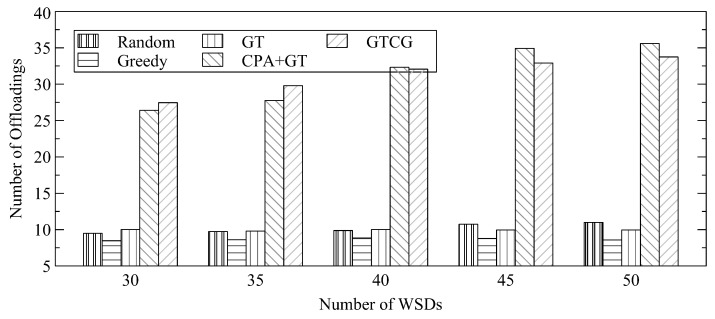
Average Number of Offloadings for Different Number of WSDs.

The number of offloadings for different numbers of WSDs is shown in Figure 3. The figure shows the difference among different approaches based on game theory with and without considering channel gain. The figure shows that if there are more channel resources for WSDs, there are more opportunities for GTCG to offload tasks to the edge server because the WSDs of GTCG can change their decisions among the channels. However, when the channel resources are scarce, the channels that GTCG can select are reduced for the WSDs with lower channel-gain values to degrade the number of offloadings. CPA+GT also does not have the problem that WSDs rob channels from WSDs with lower channel gain by changing the channel selection. It can maintain transmission performance even when the channel resources are reduced. The performance of the random decision could outperform GT because GT could allow a WSD to occupy one single channel. If the approach of the random decision could evenly allocate WSDs in different channels, the random-decision approach could achieve better performance with respect to the number of offloadings.

**Figure 4:**
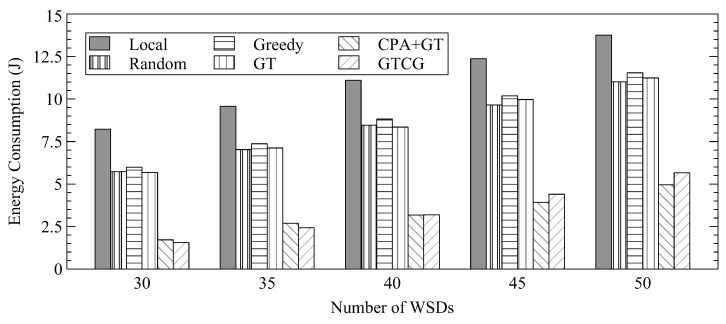
Average Energy Consumption for Different Number of WSDs.

Next, we show the overall energy consumption of WSDs in Figure 4. By offloading computation tasks to edge servers, the energy consumption of WSDs can be improved. Since GTCG and CPA+GT can offload more tasks to the edge server as shown in Figure 3, they consume less energy as compared to the other approaches.

**Figure 5:**
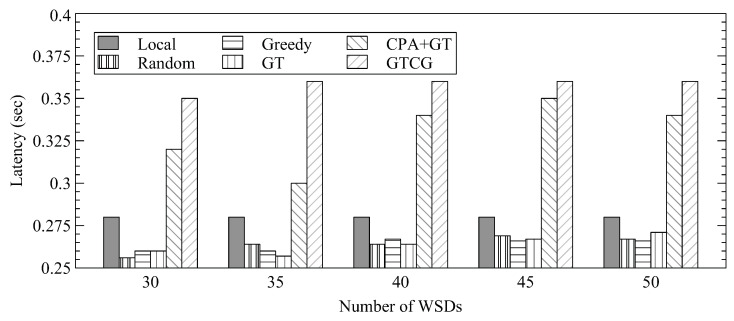
Average Latency for Different Number of WSDs.

Figure 5 shows the average latency for completing tasks. We note that all tasks can be accomplished within their time constraints since each task can always be computed locally. Both GTCG and CPA+GT have longer latency because each offloading requires additional transmission latency. Therefore, the average latency could be decreased with more local-computation tasks. The latency of CPA+GT may decrease with more WSDs because the percentage of WSDs with successful offloadings decreases. The difference between GTCG/CPA+GT and the other approaches is about 111 milliseconds or less.

**Figure 6:**
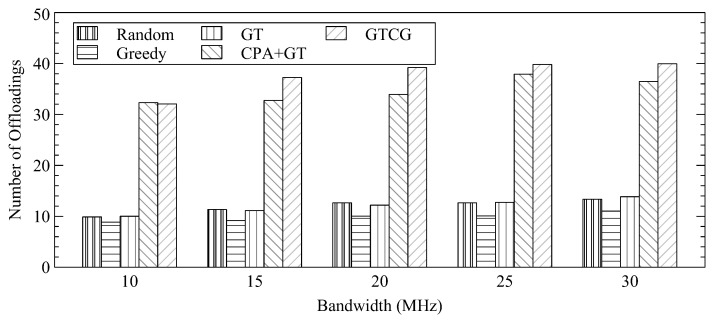
Average Number of Offloadings with Different Bandwidth.

Figure 6 shows the number of offloadings with different channel bandwidths. As the per-channel bandwidth increases, GTCG can always have a greater number of offloaded tasks as compared to CPA+GT because the additional resources cannot significantly affect the decisions of CPA+GT. Instead, GTCG allows WSDs to change their channel selections. Since the average available resources for each WSD increase, when there is a WSD to occupy another channel, the impact on the WSDs of the occupied channel would be less significant. As for other methods that do not consider channel gain, although the number has increased, it is still far from GTCG.

**Figure 7:**
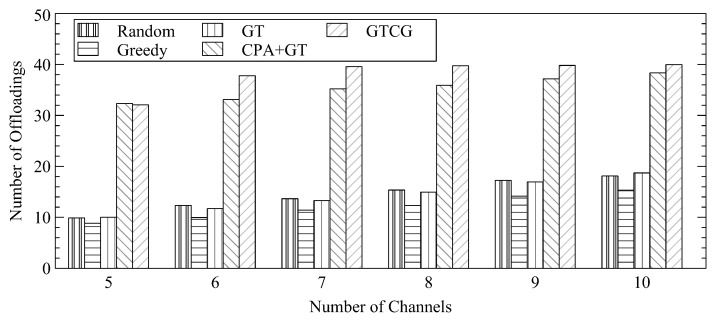
Average Number of Offloadings with Different Number of Channels.

In Figure 7, we increase the number of channels to show the average number of offloadings. Likewise, when the available resources increase, GTCG can always offload more tasks. In fact, GTCG could offload all tasks with eight channels. On the contrary, when the number of channels for CPA+GT is increased to more than 10, it is still impossible to achieve offloading for all WSDs. GTCG provides the best offloading performance among all compared algorithms.

**Figure 8:**
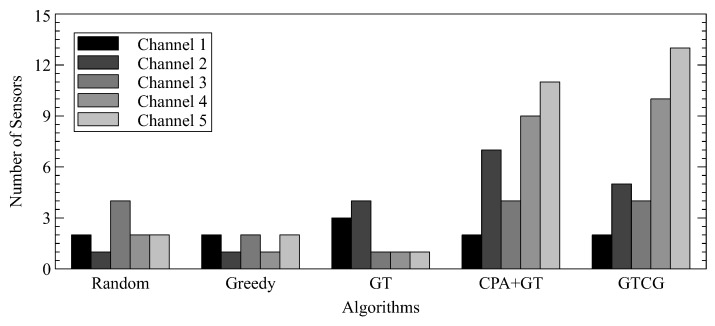
The Number of WSDs in Each Channel.

We further demonstrate the problem of a single WSD occupying a wireless channel, as mentioned earlier. Figure 8 shows the number of channels selected by the WSD in each approach. Among them, we can see that random decision, greedy and GT have one channel occupied by only one WSD to cause unfair channel allocation. The imbalance channel allocation for WSDs can be avoided by considering the channel quality.

In addition to showing the number of WSDs in each channel, we also present the standard deviation for the number of WSDs in each channel for different approaches in Table 1. Both GTCG and CPA+GT have smaller values of standard deviation as compared to the other approaches. We can thus conclude that the decisions of channel selection from the proposed approaches are better balanced. CPA+GT has a smaller value of standard deviation than GTCG because it does not allow each WSD to change their channel selections.

**Figure 9:**
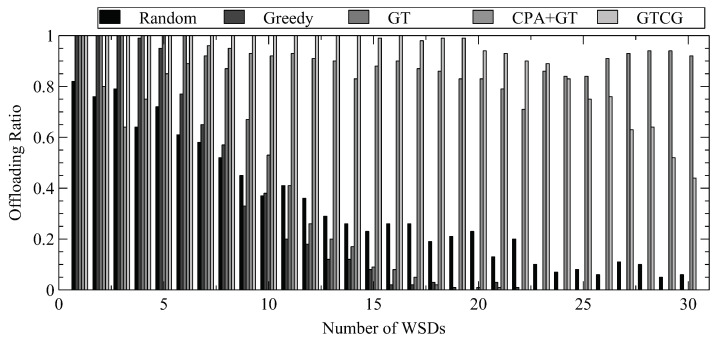
Offloading Ratios for Different Number of WSDs.

We illustrate the difference in WSD fairness between GTCG/CPA+GT and other methods by performing 100 computation tasks and show the percentage of successful offloading for each WSD in descending order of their channel gains in Figure 9. The methods without considering the channel gains of WSDs have imbalanced offloading ratios among WSDs. Moreover, the WSDs with better channel gains may not successfully offload their tasks. However, it is not the case for CTCG. When a WSD cannot acquire sufficient resources on the original channel, it may still offload its task by changing its channel selection. Although GTCG/CPA+GT cannot keep WSDs with different channel gains having the same offloading ratios, they do increase the offloading ratios for WSDs with low channel gains.

**Figure 10:**
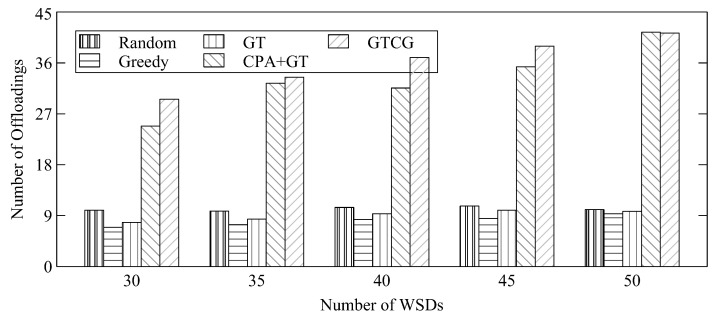
Average Number of Offloadings for Uneven WSD Distribution.

Next, we consider the case of unevenly distributed WSD locations, where 25% of WSDs are close to the basestation, and 75% of the WSDs are located at the border of the basestation coverage. Figure 10 shows that the performance of GTCG and CPA+GT is not affected by the uneven WSD distribution. In addition, GTCG always has better performance than CPA+GT because the channel pre-allocation of CPA+GT may limit the offloading decisions of some WSDs.

**Figure 11:**
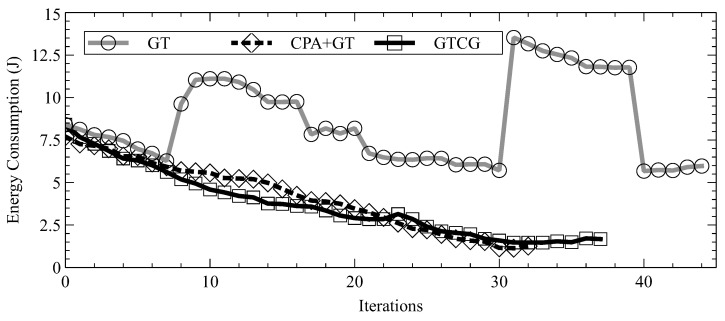
Convergence Performance.

Figure 11 presents an iterative process of three methods based on game theory. We observe that GTCG and CPA+GT reach Nash equilibrium within 32 and 37 iterations, respectively. The GT takes 44 iterations to reach convergence with severe oscillation of energy consumption. The oscillation occurs with some WSD and with better quality occupies a channel to make the original WSDs of the same channel suffer from high transmission interferences. These WSDs may change their channels to result in low convergence. CPA+GT converges faster than GTCG because CPA+GT assigns channels for each WSD initially. As a result, each update of offloading decision only affects the WSDs in the same channel. Although GTCG has a slightly slower convergence performance than CPA+GT, it achieves better offloading performance as a reasonable tradeoff.

## 7. Conclusions

In this paper, we consider the efficient computation offloading problem of multiple WSDs. As the number of WSDs increases, we consider the computation offloading problem in a multi-channel wireless sensor network for better transmission performance. However, the transmission performance of WSDs in the same channel is mainly affected by the WSDs with high channel gains. Accordingly, we propose a channel-assignment approach for WSDs. Our approach can arrange WSDs with similar channel gains in the same channel to effectively improve transmission performance. Then, we formulate a decentralized computational offloading decision problem and propose an algorithm based on game theory. We further prove the existence of Nash equilibrium for our algorithm. The simulation results show that our algorithm can effectively increase the number of offloadings by jointly considering the channel gain of each WSD for channel selection to minimize the energy consumption of WSDs. Our algorithm also has fast convergence performance. Our future work attempts to further increase the number of successful offloadings by employing heterogenous transmission techniques.

## Figures and Tables

**Table 1:** Standard Deviation for the Number of WSDs in Different Channels.

Method	Standard Deviation
Random	11.02
Greedy	12.16
GT	11.67
CPA+GT	2.56
GTCG	3.53

## Data Availability

Not applicable.
